# Effect of body tissue composition on the outcome of patients with metastatic non-small cell lung cancer treated with PD-1/PD-L1 inhibitors

**DOI:** 10.1371/journal.pone.0277708

**Published:** 2023-02-10

**Authors:** Dimitrios Makrakis, Konstantinos Rounis, Alexandros-Pantelis Tsigkas, Alexandra Georgiou, Nikolaos Galanakis, George Tsakonas, Simon Ekman, Chara Papadaki, Alexia Monastirioti, Meropi Kontogianni, Ioannis Gioulbasanis, Dimitris Mavroudis, Sofia Agelaki

**Affiliations:** 1 Department of Medical Oncology, University General Hospital, Heraklion, Crete, Greece; 2 Jacobi Medical Center, Albert Einstein College of Medicine, The Bronx, NY, United States of America; 3 Comprehensive Cancer Center, Karolinska University Hospital, Stockholm, Sweden; 4 Department of Nutrition & Dietetics, School of Health Sciences and Education, Harokopio University, Athens, Greece; 5 Department of Medical Imaging, University General Hospital, Heraklion, Crete, Greece; 6 Department of Oncology-Pathology, Karolinska Institutet, Stockholm, Sweden; 7 Laboratory of Translational Oncology, School of Medicine, University of Crete, Heraklion, Greece; 8 Department of Medical Oncology, Animus Kyanus Stavros General Clinic, Larissa, Greece; IRCCS Ospedale Policlinico San Martino, Genova, Italy, ITALY

## Abstract

Obesity and sarcopenia have been reported to affect outcomes in patients with non-small cell lung cancer (NSCLC) treated with immune checkpoint inhibitors (ICIs). We analyzed prospective data from 52 patients with non-oncogene driven metastatic NSCLC treated with ICIs. Body tissue composition was calculated by measuring the fat and muscle densities at the level of 3^rd^ lumbar vertebra in each patient computed tomography scan before ICI initiation using sliceOmatic tomovision. We converted the densities to indices [Intramuscular Fat Index (IMFI), Visceral Fat Index (VFI), Subcutaneous Fat Index (SFI), Lumbar Skeletal Muscle Index (LSMI)] by dividing them by height in meters squared. Patients were dichotomized based on their baseline IMFI, VFI and SFI according to their gender-specific median value. The cut-offs that were set for LMSI values were 55 cm^2^/m^2^ for males and 39 cm^2^/m^2^ for females. SFI distribution was significantly higher (p = **0.040**) in responders compared to non-responders. None of the other variables affected response rates. Low LSMI HR: **2.90** (95% CI: 1.261–6.667, p = **0.012**) and low SFI: **2.20** (95% CI: 1.114–4.333, p = **0.023**) values predicted for inferior OS. VFI and IMFI values did not affect survival. Subcutaneous adipose and skeletal muscle tissue composition significantly affected immunotherapy outcomes in our cohort.

## 1. Introduction

Immunotherapy (I-O) in the form of immune checkpoint inhibitors (ICIs) has significantly improved survival outcomes in the setting of a plethora of metastatic malignancies, including non-small cell lung cancer (NSCLC) [1], since it offers the possibility for durable remissions in a significant proportion of affected individuals. However, there is a scarcity of available biomarkers for the prediction of outcome in I-O treated patients with metastatic NSCLC [2]. Programmed death ligand- (PD-L)1 expression in tumor cells or immune cells of the tumor micro-environment (TME) consists the only approved biomarker thus far and its use suffers from significant limitations [3, 4].

Obesity poses one of the major health issues on a global scale [5] and it has been recognized as a risk factor for a wide range of malignancies [6]. More specifically, it has been proposed as the second most common risk factor for cancer development after tobacco exposure [7] and it has been linked with adverse treatment and survival outcomes in cancer patients [7, 8].

In contrast with these findings, large scale retrospective data have reported that obese individuals with higher Body Mass Index (BMI) values treated with ICIs for a variety of underlying malignancies experienced favorable outcomes compared to non-obese ones [9–11].

However, BMI as a marker of adipose tissue composition has limited accuracy since it is not able to distinguish between the differential fat depositions amongst different body compartments. Adipose tissue compartments are known to have substantial differences concerning their endocrine and immune properties [12, 13]. Furthermore, adipose tissue composition has been correlated with survival outcomes in cancer patients. High visceral fat percentage has been associated with poor outcomes in patients with endometrial cancer [14], whereas high subcutaneous fat density has been associated with favorable outcomes among patients with prostate, colorectal, and renal cancer [15, 16].

In addition, skeletal muscle depletion has been consistently associated with adverse outcomes in cancer patients across several studies [17–19] and serves as a criterion for the definition of cancer cachexia syndrome [20]. Recently, low skeletal muscle density has been reported as a negative predictive and prognostic factor in patients with metastatic melanoma [21] and NSCLC [22, 23] receiving treatment with ICIs. Finally, the presence of low muscle mass density was associated with increased toxicities from ipilimumab administration in individuals with metastatic melanoma [24].

Based on the above data we hypothesized that adipose and skeletal muscle tissue composition influences I-O outcomes in cancer patients. In order to further test our hypothesis, we analyzed clinical and radiological data from patients with metastatic NSCLC that received treatment with ICIs at the University Hospital of Heraklion, Crete from 2017 to 2020.

## 2. Materials and methods

### 2.1 Patient selection

We analyzed prospective clinical and radiological data which were acquired from a prospective observational study at the University Hospital of Heraklion, Crete (ID: 2644). Our population sample consisted of patients with non-oncogene driven metastatic NSCLC that were treated with ICIs either as monotherapy or in combination with chemotherapy according to ESMO guidelines [25]. Individuals with *EGFR* mutations or *ALK* translocations were excluded before the initial screening. Our study was approved by the ethical board of the University Hospital of Heraklion, Crete (ID: 2644) and was conducted according to principles of the declaration of Helsinki. Written informed consent was obtained from all patients before enrollment.

### 2.2 Body tissue composition assessment

**Fig 1:**
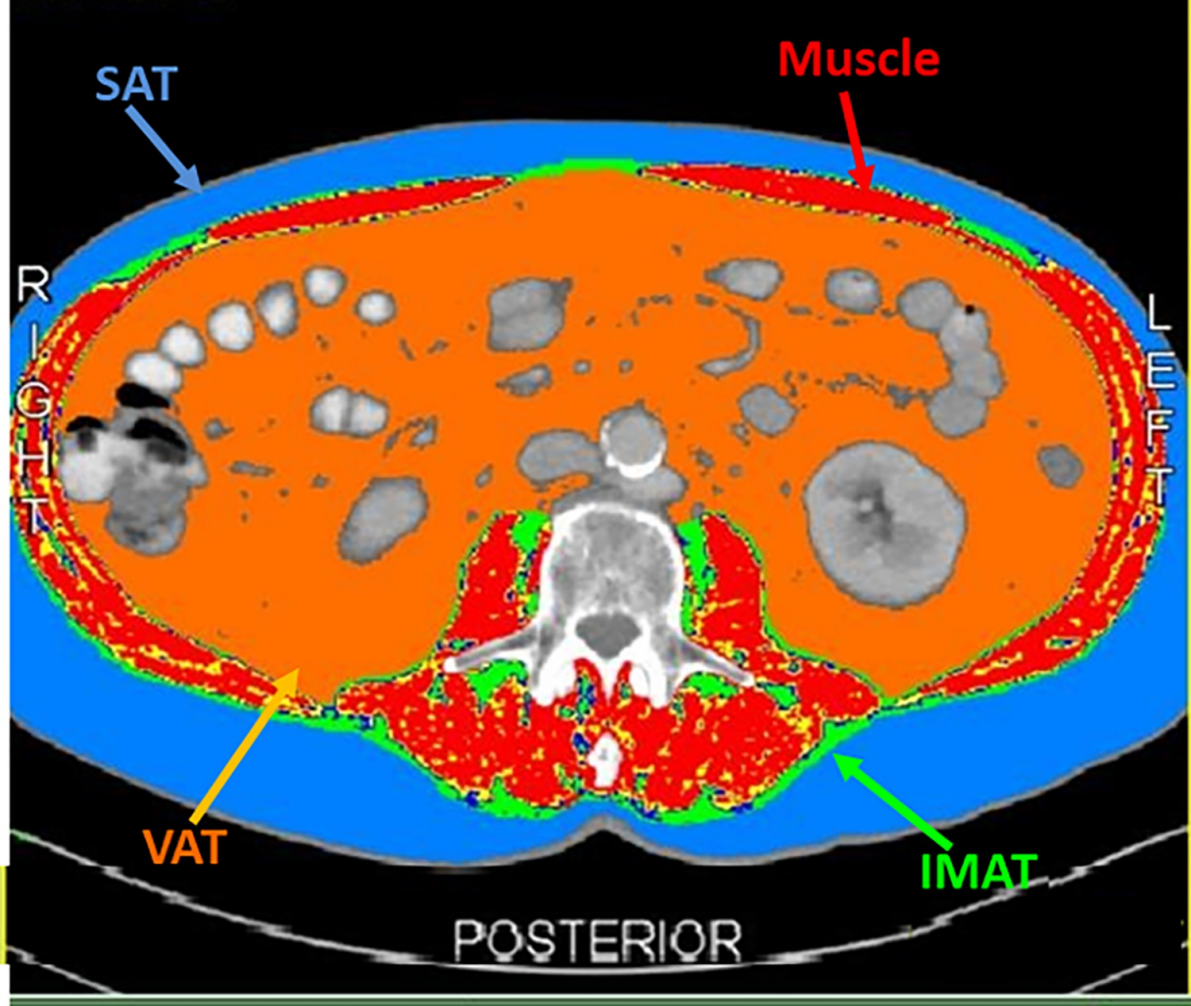
SliceOmatic tomovision analysis of the computed tomography scan of a male patient distinguishing the different context of Visceral Adipose Tissue (VAT), Intramuscular Adipose Tissue (IMAT), Subcutaneous Adipose Tissue (SAT) and skeletal muscle according to their differential Hounsfield Units (HU) references.

For the assessment of body tissue composition we analyzed images from the abdominal Computed Tomography (CT) scans of the patients before the initiation of ICIs at the level of 3^rd^ lumbar vertebra (L3) [26]. We analyzed the images using slice-o-matic tomovision software (sliceOmatic 5.0 Rev-9 Alberta Protocol). The densities of the different adipose tissue compartments and skeletal muscle were calculated through the application of differential Hounsfield Unit (HU) threshold references for each tissue compartment, respectively (−190 HU to −30 HU for intramuscular fat, −150 HU to −50 HU for visceral fat, −190 HU to −30 HU for subcutaneous fat, -29 HU to +150 for skeletal muscle) (Fig 1) [27, 28]. The fat densities (in cm^2^) and muscle density (in cm^2^) for each individual were converted to indices (in cm^2^/m^2^) by dividing them by height in meters squared (Intramuscular Fat Index: IMFI, Visceral Fat Index: VFI, Subcutaneous Fat Index: SFI and Lumbar Skeletal Muscle Index: LSMI).

We categorized the patients in a binary fashion according to their baseline IMFI, VFI and SFI based on the gender specific median value of each perspective index. Patients with baseline IMFI, VFI and SFI values below median were classified as low and those with above median values were classified as high, respectively. Patients were categorized according to their baseline LSMI according to the cut-offs for skeletal muscle depletion which were set by Fearon et al. [20]. They consist of 55 cm^2^/m^2^ for males and 39 cm^2^/m^2^ for females. Individuals with LSMI below the aforementioned cut-offs were categorized as < Lower Normal Limit (LNL) and the rest as ≥ LNL.

### 2.3 Clinical data collection

The following clinical variables: age, gender, smoking status, ECOG performance status (PS), baseline BMI, histology, organs affected with metastases, PD-L1 status and line of treatment of ICI administration were analyzed. Individuals were classified in a dichotomous fashion based on their age (<70 years old vs ≥ 70 years old), gender (male vs female), PS (0–1 vs 2), smoking status (smokers or former smokers vs non-smokers), line of treatment of ICI administration (1^st^ line vs 2^nd^ or later lines of treatment), presence of brain, liver or bone metastases, histology (squamous vs non-squamous), PD-L1 status (<1% vs ≥ 1%) and baseline albumin levels (<3.5 g/dl vs ≥ 3.5 g/dl). The cut-off value that was set for baseline albumin levels was 3.5 gram (g)/ deciliter (dl) which represents the lower normal limit in our laboratory.

We classified patients in our cohort according to their baseline BMI in a trichotomous fashion, those with BMI < 25 kg/m^2^, overweight individuals with BMI values ≥ 25 kg/m^2^ but < 30 kg/m^2^ and obese with a BMI > 30 kg/m^2^.

Grade 3 or 4 immune related adverse events (irAEs) were recorded according to ESMO guidelines [29].

#### 2.3.1 Response and survival outcomes assessment

Response assessment to treatment was conducted according to RECIST 1.1 criteria [30]. Patients were categorized according to their best response to ICIs as having complete response (CR), partial response (PR), stable disease (SD) and progressive disease (PD). Patients that had achieved CR or PR were classified as responders and the rest as non-responders. Objective response rate (ORR) was defined as the percentage of individuals who achieved CR or PR as best response to treatment. Patients were also categorized as achieving disease control (DC) if they had experienced CR or PR or SD as best response to treatment. We also compared response SFI scores both between patients with vs without disease control (defined as CR, PR or SD) and between patients with or without response (defined as CR or PR). This approach allowed for more detailed presentation of findings.

Progression-free survival (PFS) was calculated from the initiation of ICI until the date of disease progression or death. Overall survival (OS) was calculated from the beginning of ICI until the date of death. Individuals who had not progressed or were still alive at the time of data analysis were censored at the date of last follow-up.

#### 2.3.2 Statistical analysis

Statistical analyses were performed with SPSS 25.0.0 software (IBM Corp., Armonk, NY, USA). Descriptive statistics were applied to define and analyze nominal and categorical variables. Statistical significance was set at < 0.05. Spearman’s rank correlation coefficient was applied to examine any potential correlations between BMI values with IMFI, VFI, SFI and LSMI and between the fat indices with LSMI. Mann-Whitney U test was used to examine any potential differences amongst the distributions of IMFI, VFI, SFI and LSMI values between responders and non-responders and amongst those who achieved disease control as best response to I-O vs those who experienced disease progression. Chi-square test was applied to investigate any potential associations of the analyzed categorical parameters with ORR.

The Kaplan-Meier method was applied in order to investigate the effect of the studied parameters on PFS and OS. Curves were compared with the log-rank test. We performed initially a univariate Cox regression analysis to examine the effect of BMI, IMFI, VFI, SFI and LSMI as continuous nominal variables on PFS and OS using gender as a stratification factor. In addition, a univariate Cox regression analysis was performed to calculate the Hazard Ratios (HR) of age ≥ 70 years old, PS = 2, squamous histology, bone metastases, liver metastases, brain metastases, PD-L1 < 1%, BMI < 25 kg/m^2^, low IMFI, low VFI, low SFI and LSMI < LNL on PFS and OS. We did not perform a multivariate analysis on the values that had reached statistical significance in the univariate analysis due to low number of events and small statistical sample.

A sample size and power calculation was not conducted because at the time of the initiation of data collection there were no published reports on the effect of adipose or muscle tissue composition on the outcome of patients with malignancies receiving immunotherapy. Due to the lack of available statistical information on which to base the calculations for power analysis, it would have been of no value in this exploratory, hypothesis generated study.

## 3. Results

### 3.1 Patient characteristics

A total of 52 patients were included in the analysis. Individual patient characteristics are depicted in Table 1. Median follow-up time was 9.9 months. Forty three patients (82.7%) were male and median age was 68 years old (range: 39–81 years). Forty three (82.7%) patients had received I-O as second-line treatment and 9 as a first line. All the patients that received immunotherapy as second line of treatment had previously progressed on a platinum doublet regimen. Fifty (96.2%) patients received ICIs as monotherapy and the other two in combination with chemotherapy. Objective Response Rate (ORR) was 23.1% in our cohort and 7 (13.5%) patients experienced grade 3 or 4 immune related adverse events (irAEs) as a result of I-O administration.

**Table 1:** Baseline (At the beginning of immunotherapy) patient characteristics.

	All patients	
Variable	N	%
Number of patients	52	
**Age (years)**		
Median (range)	68 (39–81)	
**Gender**		
Male	43	82.7
Female	9	17.3
**Performance status**		
0–1	41	78.8
2	11	21.2
**Smoking status**		
Active or former smokers	48	92.3
Never smokers	4	7.7
**Histology**		
Squamous	22	42.3
Non-squamous	30	57.7
**Mean baseline BMI (SD)**	26.67 (4.39)	
**Baseline BMI**		
< 25 kg/m^2^	18	34.6
25 kg/m^2^ ≤ BMI < 30 kg/m^2^	21	40.4
BMI > 30 kg/m^2^	13	25
**Brain metastases**		
Yes	10	19.2
No	42	80.8
**Liver metastases**		
Yes	14	26.9
No	38	73.1
**Bone metastases**		
Yes	15	28.8
No	37	71.2
**Baseline albumin levels**		
≥3.5 g/dl	41	78.8
<3.5 g/dl	6	11.5
Missing values	5	9.6
**PD-L1 levels**		
< 1%	10	19.2
1% < PD-L1 < 50%	15	28.8
≥ 50%	7	13.5
Missing values	20	38.5
**Line of treatment of ICI administration**		
1^st^ line	9	17.3
2^nd^ line	43	82.7
**Immunotherapy agent**		
Nivolumab	34	65.4
Pembrolizumab	16	30.8
Atezolizumab	2	3.8
**Mode of ICI administration**		
Monotherapy	50	96.2
Combination with chemotherapy	2	3.8
**Baseline LSMI**		
< LNL	16	30.8
≥ LNL	36	69.2
**Median baseline IMFI (cm** ^**2**^ **/m** ^**2**^ **)**		
Males (N = 43) (range)	9.87 (3.53–35.13)	
Females (N = 9) (range)	10.52 (4.24–39.45)	
**Median baseline VFI (cm** ^**2**^ **/m** ^**2**^ **)**		
Males (N = 43) (range)	45.15 (6.34–172.82)	
Females (N = 9) (range)	31.20 (12.78–92.75)	
**Baseline SFI (cm** ^**2**^ **/m** ^**2**^ **)**		
Males (N = 43) (range)	50.73 (4.61–136.65)	
Females (N = 7) (range)	55.36 (44.24–149.26)	

**Abbreviations:** BMI = Body mass index, SD = Standard deviation, PD-L1 = Programmed death ligand-1, ICI = Immune checkpoint inhibitor, LSMI: Lumbar skeletal muscle index (At the level of 3^rd^ lumbar vertebra), LNL: Lower normal limit, 55 cm^2^/m^2^ for males and 39 cm^2^/m^2^ for females, IMFI = Intramuscular Fat Index (At the level of 3^rd^ lumbar vertebra), VFI = Visceral Fat Index (At the level of 3^rd^ lumbar vertebra), SFI = Subcutaneous Fat Index (At the level of 3^rd^ lumbar vertebra)

Mean baseline body mass index (BMI) was 26.67 kg/m^2^. Thirteen patients (25%) were classified as obese with BMI > 30 kg/m^2^, 21 patients (40.4%) had BMI values ≥ 25 kg/m^2^ but < 30 kg/m^2^, and the rest 34.6% of patients had a BMI < 25 kg/m^2^. The median values for intramuscular fat index (IMFI), visceral fat index (VFI) and subcutaneous fat index (SFI) for males and females, respectively, are demonstrated in Table 1. Thirty-six (69.2%) patients were categorized as sarcopenic with lumbar skeletal muscle index (LSMI) values < lower normal limit (LNL).

VFI (rho = 0.810, p = <0.001) (S1A Fig in S1 File), SFI (rho = 0.623, p = <0.001) (S1B Fig in S1 File) and LSMI (rho = 0.429, p = 0.002) (S1C Fig in S1 File) showed a significant positive correlation with BMI values whereas IMFI (rho = 0.242, p = 0.084) did not (S1D Fig in S1 File). In addition, IMFI was not correlated with LSMI (rho = - 0.172, p = 0.222) (S2A Fig in S1 File). On the contrary, VFI (rho = 0.466, p = 0.001) (S2B Fig in S1 File) and SFI (rho = 0.289, p = 0.042) (S2C Fig in S1 File) were positively correlated with LSMI.

### 3.2 Response assessment

**Fig 2:**
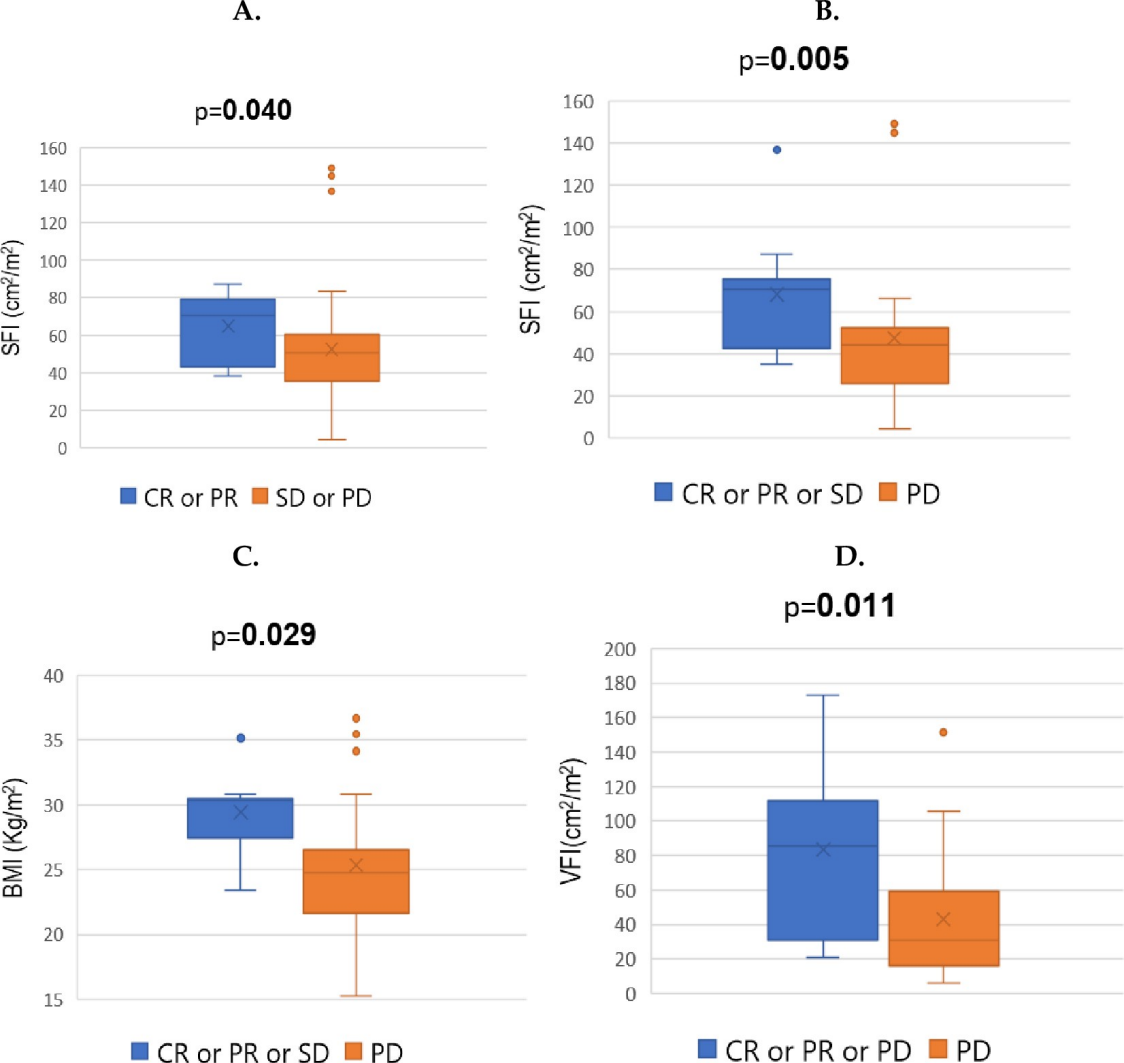
Box-plots depicting the baseline* differential distributions (Mann Whitney U test) of A. SFI (cm^2^/m^2^) between responders (CR or PR) and non-responders (SD or PD) to I-O B. SFI (cm^2^/m^2^) between patients who achieved disease control (CR or PR or SD) as result of I-O versus those who developed disease progression (PD) C. BMI (kg/m^2^) between patients who achieved disease control as result of I-O administration in comparison to those who developed disease progression and D. VFI (cm^2^/m^2^) between individuals who experienced disease control under I-O versus those who had disease progression. **Abbreviations:** I-O = Immunotherapy; BMI = Body mass index; VFI = Visceral Fat Index (At the level of 3^rd^ lumbar vertebra); SFI = Subcutaneous Fat Index (At the level of 3^rd^ lumbar vertebra); CR = Complete response; PR = Partial response; SD = Stable disease, PD = Progressive disease. *Baseline = At the beginning of immunotherapy.

The distributions of BMI (p = 0.391), IMFI (p = 0.688), VFI (p = 0.460) and LSMI (p = 0.501) did not differ significantly between responders and non-responders to I-O (S3A-S3D Fig in S1 File). Responders had higher SFI values in comparison to non-responders at a statistically significant level (p = **0.040**) (Fig 2A).

In addition, individuals who achieved disease control had higher SFI values (p = **0.005**) (Fig 2B), higher BMI values (p = **0.029**) (Fig 2C) and higher VFI values (p = **0.011**) (Fig 2D) at a statistically significant level in comparison to patients who demonstrated disease progression as best response to treatment. IMFI values (p = 0.164) and LSMI values (p = 105) did not differ significantly among the individuals who demonstrated disease control vs those who experienced PD (S3E and S3F Fig in S1 File). None of the analyzed categorical parameters affected ORR at a statistically significant level (S1 Table in S1 File). Information on response to ICIs, rate of Grade 3–4 irAEs and patient survival (median progression free survival, median overall survival) can be found on Table 2.

**Table 2:** Treatment and response characteristics.

	All patients	
Variable	N	%
**Response to ICIs**		
CR	1	1.9
PR	11	21.2
SD	15	28.8
PD	25	48.1
**Grade 3–4 irAEs**		
Yes	7	13.5
No	45	86.5
**Progression-free survival (months)**		
Median (95% CI)	4.67 (3.53–5.81)	
**Overall survival (months)**		
Median (95% CI)	10.33 (6.83–13.84)	
**Follow-up (months)**		
Median (95% CI)	9.90 (5.07–14.73)	

**Abbreviations:** CR: Complete response, PR: Partial response, SD: Stable disease, PD: Progressive disease, irAEs = Immune-related Adverse Events

### 3.3 Survival outcomes

**Fig 3:**
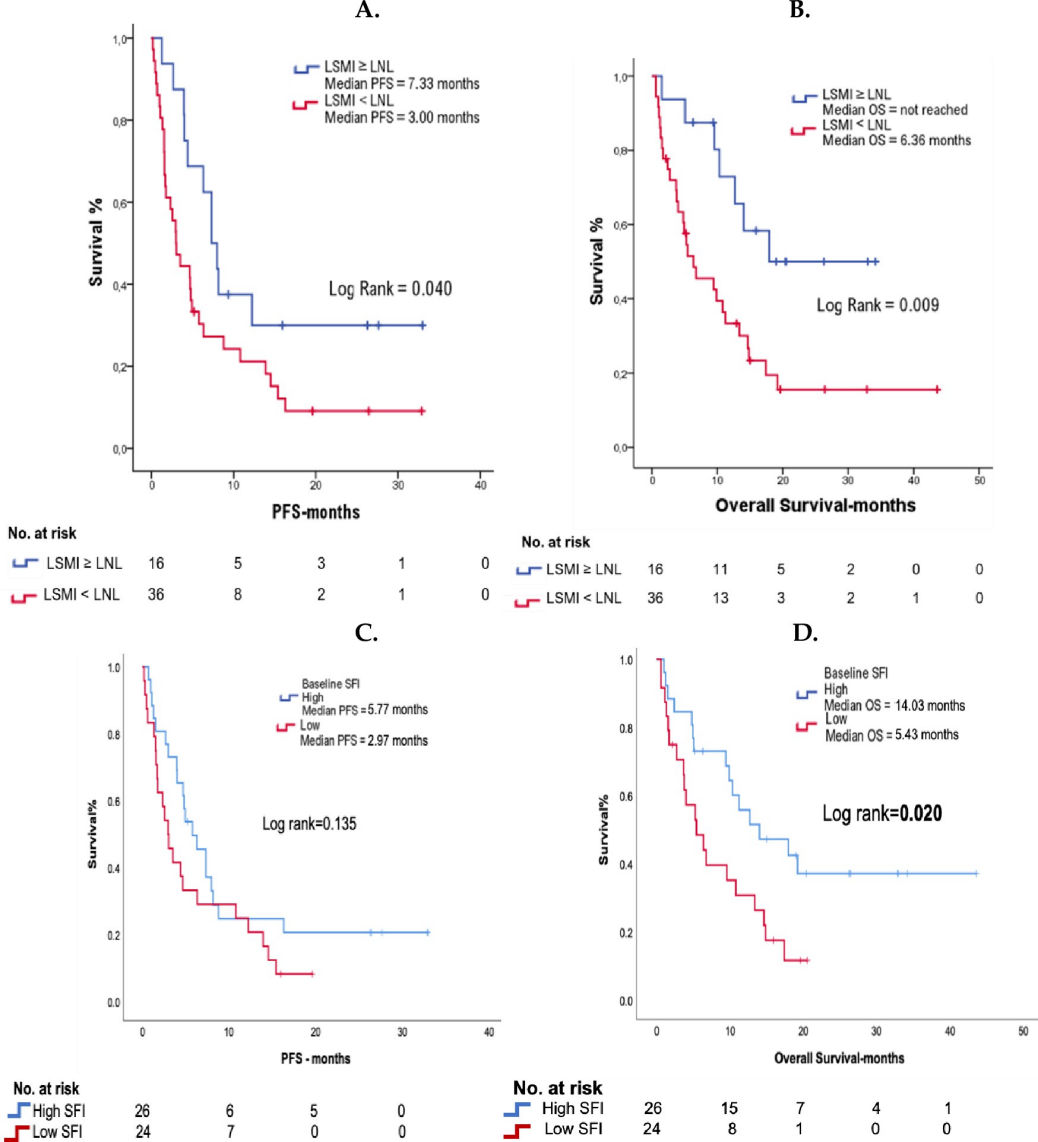
Kaplan-Meier curves demonstrating the effect of A. Baseline*^1^ LSMI*^2^ values on PFS B. Baseline LSMI values on OS C. Baseline SFI*^3^ values on PFS D. Baseline SFI values on OS. **Abbreviations:** LSMI = Lumbar skeletal muscle index (At the level of 3^rd^ lumbar vertebra); SFI = Subcutaneous Fat Index (At the level of 3^rd^ lumbar vertebra); OS = Overall survival; PFS = Progression free survival. *^1^ **Baseline:** At the beginning of immunotherapy with PD-1/PD-L1 inhibitors. *^2^ **LNL:** Lower normal limit, 55 cm^2^/m^2^ for males and 39 cm^2^/m^2^ for females. *^3^ High and low classification for SFI represents above and below gender specific median value, respectively.

The effect of the studied variables on PFS and OS is summarized in S2 Table in S1 File. Patients with baseline LSMI < LNL experienced inferior PFS (3.30 vs 7.33 months, p = **0.040**) (Fig 3A) and OS (6.37 vs not reached months, p = **0.009**) (Fig 3B), respectively. Low SFI did not affect PFS (2.97 vs 5.77 months, p = 0.135) (Fig 3C) but it was negatively associated with OS (5.43 vs 14.03 months, p = **0.020**) (Fig 3D) at a statistically significant level. In addition, the presence of brain metastases demonstrated a significant association with inferior PFS (1.57 vs 4.93 months, p = **0.006**) but not OS (4.80 vs 12.70 months, p = 0.083). Albumin levels < 3.5 g/dl were associated with inferior PFS (1.70 vs 4.80 months, p = **0.011**) and OS (1.70 vs 11.23 months, p = **0.001**). None of the other analyzed parameters demonstrated any significant association with PFS or OS (S2 Table in S1 File).

The subgroup analysis investigating the effect of the combination of SFI and LSMI values on survival outcomes demonstrated that the three subgroups that were created differed significantly in terms of OS (p = **0.004**) (S4A Fig in S1 File). However, survival outcomes did not differ significantly between patients with high SFI and LSMI < LNL and patients with both high SFI and LSMI ≥ LNL (9.90 vs 17.93 months, p = 0.285) (S4B Fig in S1 File).

IMFI, VFI, SFI, LSMI and BMI as continuous nominal variables did not demonstrate any significant association with PFS (Table 3). However, SFI values exhibited a positive association with improved survival HR = **0.983** (95% CI: 0.970–0.987, p = **0.014**) (Table 3 and S5 Fig in S1 File). None of the other body composition indices as a continuous variable was associated with OS at a statistically significant level.

**Table 3:** Univariate analysis using Cox regression method investigating the hazard ratios of the BMI*, IMFI, VFI and SFI as continuous nominal variables (cm^2^/m^2^) on PFS and OS. Gender was used as a stratification factor.

COX REGRESSION	PFS		OS	
UNIVARIATE ANALYSIS	HR (95% Confidence Intervals)	p value	HR (95% Confidence Intervals)	p value
BMI (kg/m^2^)	0.973 (0.895–1.059)	0.528	0.936 (0.853–1.028)	0.165
LSMI (cm^2^/m^2^)	0.981 (0.950–1.013)	0.236	0.973 (0.941–1.006)	0.102
IMFI (cm^2^/m^2^)	0.996 (0.955–1.039)	0.866	0.950 (0.890–1.014)	0.121
VFI (cm^2^/m^2^)	0.998 (0.989–1.007)	0.646	0.991 (0.980–1.002)	0.095
SFI (cm^2^/m^2^)	0.993 (0.982–1.005)	0.246	0.983 (0.970–0.997)	**0.014**

**Abbreviations:** BMI: Body mass index (kg/m^2^), LSMI: Lumbar skeletal muscle index (cm^2^/m^2^), IMFI = Intramuscular fat index (cm^2^/m^2^), VFI = Visceral fat index (cm^2^/m^2^), SFI = Subcutaneous fat index (cm^2^/m^2^)

* BMI, IMFI, VFI and SFI were calculated at the beginning of immunotherapy

**Fig 4:**
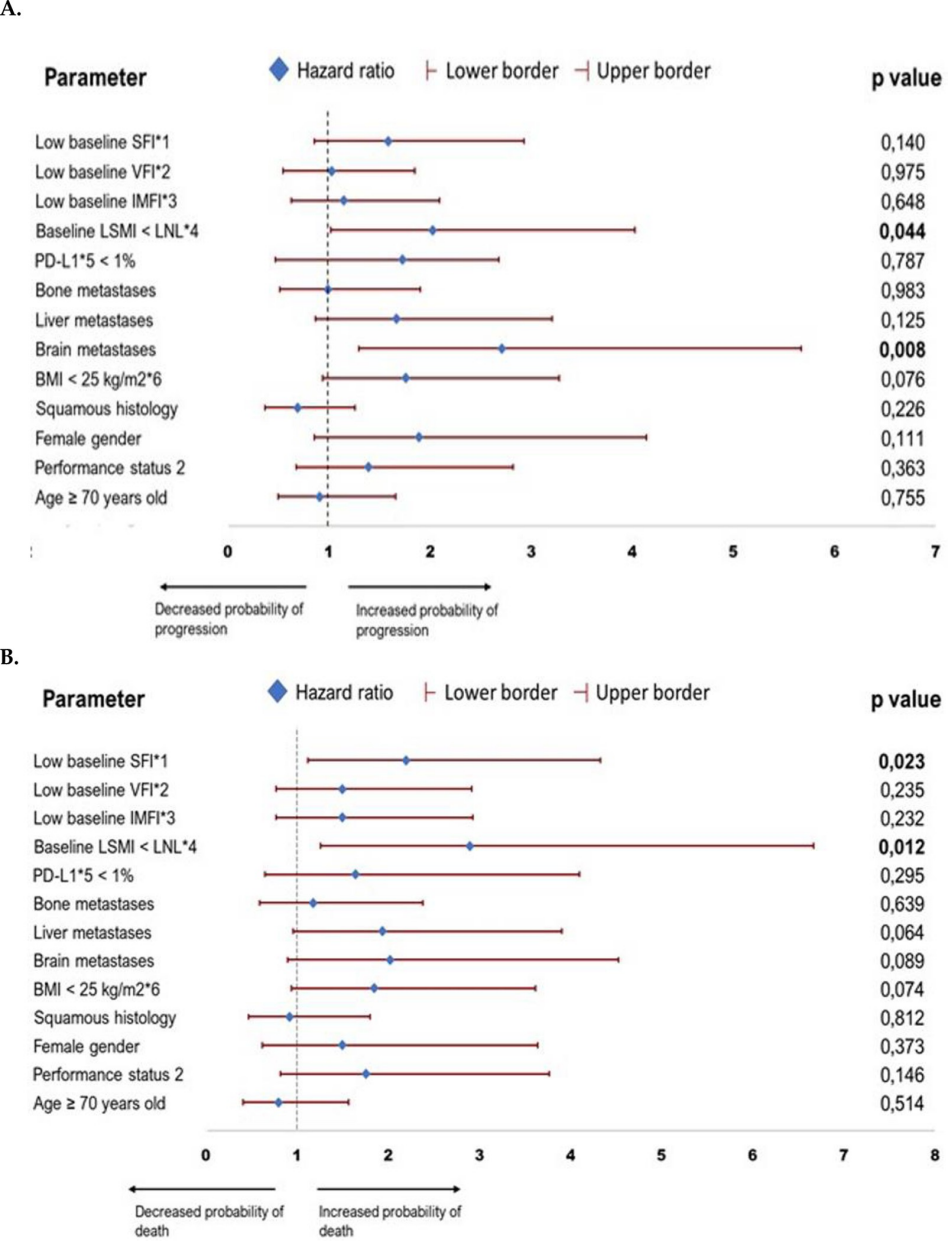
Forest plots demonstrating the hazard ratios and their 95% confidence intervals of the analyzed parameters on A. Probability of disease progression B. Probability of death under treatment with ICIs. **Abbreviations:** BMI = Body mass index; PD-L1 = Programmed death ligand-1, ICI = Immune checkpoint inhibitor; LSMI: Lumbar skeletal muscle index (At the level of 3^rd^ lumbar vertebra); LNL: Lower normal limit, 55 cm^2^/m^2^ for males and 39 cm^2^/m^2^ for females; IMFI = Intramuscular Fat Index (At the level of 3^rd^ lumbar vertebra); VFI = Visceral Fat Index (At the level of 3^rd^ lumbar vertebra); SFI = Subcutaneous Fat Index (At the level of 3^rd^ lumbar vertebra). *^1,2,3,4,5,6^ SFI, VFI, IMFI, LSMI, PD-L1 and BMI values were calculated at the beginning of treatment with ICIs. *^1,2,3^ Low for SFI, VFI and IMFI means below gender specific median value.

The presence of brain metastases HR = **2.71** (95% CI:1.299–5.667, p = **0.008**) and baseline LSMI < LNL HR = **2.03** (95% CI: 1.018–4.032, p = **0.044**) were the only two parameters that predicted for increased probability of disease progression (Fig 4A and S3 Table in S1 File). In the univariate analysis for OS baseline LSMI < LNL HR = **2.90** (95% CI:1.261–6.667, p = **0.012**) and low SFI HR = **2.20** (1.114–4.333, p = **0.023**) predicted for inferior survival (Fig 4B and S3 Table in S1 File).

## 4. Discussion

In this study we demonstrated that reduced subcutaneous adiposity and skeletal muscle depletion could possibly constitute negative prognostic factors for individuals with metastatic NSCLC treated with ICIs. In our cohort, low SFI values (either as a continuous variable or as a categorical variable) were significantly associated with inferior survival outcomes. In addition, responders to treatment exhibited higher SFI distributions in comparison to non-responders.

Several clinical studies on cancer patients treated with ICIs have demonstrated that high BMI values are associated with favorable outcomes [9–11]. In a retrospective study of patients with clear cell renal carcinoma, obese patients were found to have increased peritumoral adipose tissue inflammation and better survival outcomes [31]. Martini et al. [32] performed a retrospective analysis of 90 patients that had received immunotherapy for a wide range of malignancies in the context of phase I trials and demonstrated that high SFI/IMFI ratio consisted an independent predictor of superior OS. On the contrary, Woodall et al. [33] investigated the effect of BMI and body composition on treatment outcomes in melanoma patients receiving ICIs. They reported that high total visceral and subcutaneous adipose tissue index was associated with reduced PFS [33]. However, when they investigated the effect of subcutaneous adipose tissue index it was not associated with reduced PFS or OS [33]. Finally Schols et al. [34] (citation: 10.1002/jcsm.12698) demonstrated in a cohort of 106 NSCLC patients treated with nivolumab that the weight loss > 2% under treatment which was reflected with a significant loss in SFI and VFI consisted a negative prognostic survival factor. Our results are consistent with the results of Martini et al. [32] demonstrating a potential positive link between increased subcutaneous adiposity and improved survival outcomes. The novelty of our findings is that we demonstrate a potential link between baseline SFI values and clinical outcomes in a population with homogeneous underlying malignancy histology. The fact that we were not able to demonstrate any association between BMI and immunotherapy outcomes can be partially attributed to the small population sample since overweight/obese patients in our cohort had better outcomes but this difference did not reach statistical significance. The discordance between our results and the results from Woodall et al. [33] can be explained by selection bias among patient populations, different underlying malignancies and different immunotherapies.

However, our findings along with the results of the majority of the aforementioned studies could suggest a link between adiposity and improved antitumor immune response that merits further evaluation. There are insufficient data for the proposal of a robust biological link, but subcutaneous adipose tissue is the compartment responsible for the production of leptin [35]. Leptin was demonstrated by Wang et al. [36] to be at least partially responsible for the effects of PD-1 upregulation and immune aging in obese mice as a counterbalance mechanism for the inflammatory status that accompanies obesity. However, the same biological effect may be responsible for the increased sensitivity to PD-1 inhibition in obese mice and humans, since they rely on PD-L1 axis as a feedback mechanism for the immunologic equilibrium of their underlying inflammatory process and the release of this inhibition might result in more effective antitumor responses. On the other hand, it is unclear whether the link between high SFI and improved outcomes is causal and not an epiphenomenon due to a potential underlying cancer cachexia syndrome and a subsequent process of browning of white adipose tissue [37].

Furthermore, there is accumulation of evidence on the cellular pathways that connect adipose tissue composition and immune system regulation [38]. Adipose tissue serves as a nest for a plethora of immune cells such as macrophages, CD4+ T cells, CD8+ T cells, T regulatory (Treg) cells, iNKT cells and γδ T cells [39]. The infiltration of macrophages into the adipose tissue of obese mice has been reported to switch them from the M2 phenotype to M1 proinflammatory phenotype, a phenomenon that has not been observed in non-obese mice [40, 41]. Furthermore, the adipose tissue of obese mice was found to be infiltrated by increased numbers of effector T cells and to demonstrate a high CD8+/CD4+ ratio with diminished number of Tregs [42]. Tregs act as a negative regulator of the inflammatory process in the adipose tissue of normal weight mice, but their numbers are greatly reduced in the adipose tissue of obese mice [43]. Based on these experimental data it is possible that obese individuals are more susceptible to checkpoint inhibition in the setting of underlying malignancies, due to an underlying pro-inflammatory status characterized by increased Th1 responses, macrophage polarization to an M1 phenotype and reduced number of Tregs in their adipose tissue reservoirs. However, this hypothesis needs to be further tested with additional preclinical and translational data.

LSMI levels consistent with sarcopenia were also an adverse prognostic factor in our cohort. Sarcopenia has been linked to adverse outcomes in cancer patients in multiple studies [17, 18] before the introduction of ICIs. In addition, it is one of the criteria for the definition of cancer cachexia, which consists one of the most well recognized adverse prognostic factors in cancer patients [20]. Our results are in accordance with other studies on the role of skeletal muscle depletion as a negative prognostic and predictive factor in ICI treated cancer patients [19, 21, 22, 34, 44].

In addition, we performed a subgroup analysis to examine the survival outcomes of patients with both low adiposity and muscle depletion in comparison to the patients with high adiposity but muscle depletion and the individuals without muscle depletion and high adiposity. Patients with both low SFI and sarcopenia had the worst outcome whereas the other two subgroups did not differ significantly. Woodall et al. [33] reported that patients with adiposity and reduced muscle mass had worse outcomes. This discordance might be due to the small sample size in our cohort that can hinder possible statistically significant correlations. Nevertheless, further research is required in order to redefine the prognostic importance of sarcopenic obesity in the era of immunotherapy.

To our knowledge this is first analysis of prospective data in NSCLC patients who are treated with ICIs which investigates the effect of fat and muscle tissue composition on treatment outcomes. Our study consists of a population with a certain degree of homogeneity since the vast majority of the NSCLC patients received immunotherapy as a monotherapy with PD-1/PDL-1 inhibitors and most of the patients received it as 2^nd^ line treatment.

The major limitation of our study is the small statistical sample with limited statistical power. Due to the small sample size, we analyzed together patients treated with ICIs in the 1^st^ and 2^nd^ line setting, while we also included two patients treated with chemotherapy-ICI combination; we consider this number too small to significantly affect results. In addition, due to low number of events in the subgroup of patients with LMSI values not consistent with sarcopenia we were not able to perform a multivariate analysis for overall survival. Our sample group was treated mostly with 2^nd^ line PD-1/PDL-1 inhibitors, however a significant number of patients received them as 1^st^ line. In addition, our cohort was imbalanced according to gender with the majority of our patients being male and only a small proportion being female. Fat tissue compositions can differ significantly between the two genders and an imbalanced population can hinder significant statistical associations. Moreover, age is one of the most significant factors that affects body composition and in this study patients from all age groups were included. A power calculation was not feasible mainly due to the exploratory nature of this study and secondary because of the lack of previous publications on the effect of adipose tissue composition in ICI treated NSCLC patients in which to base an initial power calculation. Finally, due to the lack of a specified cut-off for IMFI, VFI and SFI we arbitrarily chose as a cut-off point the gender specific median value of each perspective variable.

## 5. Conclusions

Our findings demonstrate that subcutaneous adipose tissue and muscle tissue composition could be associated with outcomes with ICI treatment in NSCLC patients. Validation of these results in larger cohorts is required.

The results from our study and from similar published articles propose a potential link between subcutaneous adiposity and sensitivity to PD-1/PD-L1 inhibition. Further research on preclinical and translational lever is required to further decipher a potential association between adiposity composition and immune system function towards the finding of novel drug targets or novel biomarkers.

## Supporting information

S1 File (DOCX)
