# Correction: Effect of body tissue composition on the outcome of patients with metastatic non-small cell lung cancer treated with PD-1/PD-L1 inhibitors

**DOI:** 10.1371/journal.pone.0294384

**Published:** 2023-11-09

**Authors:** Dimitrios Makrakis, Konstantinos Rounis, Alexandros-Pantelis Tsigkas, Alexandra Georgiou, Nikolaos Galanakis, George Tsakonas, Simon Ekman, Chara Papadaki, Alexia Monastirioti, Meropi Kontogianni, Ioannis Gioulbasanis, i Mavroudis, Sofia Agelaki

An additional affiliation is missing for the second author. Konstantinos Rounis is also affiliated with: Laboratory of Translational Oncology, Medical School, University of Crete.

The correct affiliations are as follows: Dimitrios Makrakis^**1,2**^, Konstantinos Rounis^**1,3,4**^, Alexandros-Pantelis Tsigkas^**5**^, Alexandra Georgiou^**5**^, Nikolaos Galanakis^**6**^, George Tsakonas^**3,7**^, Simon Ekman^**3,7**^, Chara Papadaki^**4**^, Alexia Monastirioti^**4**^, Meropi Kontogianni^**5**^, Ioannis Gioulbasanis^8^, Dimitris Mavroudis^**1,4**^, Sofia Agelaki^**1,4**^

**1** Department of Medical Oncology, University General Hospital, Heraklion, Crete, Greece, **2** Jacobi Medical Center, Albert Einstein College of Medicine, The Bronx, NY, United States of America, **3** Comprehensive Cancer Center, Karolinska University Hospital, Stockholm, Sweden, **4** Laboratory of Translational Oncology, School of Medicine, University of Crete, Heraklion, Greece, **5** Department of Nutrition & Dietetics, School of Health Sciences and Education, Harokopio University, Athens, Greece, **6** Department of Medical Imaging, University General Hospital, Heraklion, Crete, Greece, **7** Department of Oncology-Pathology, Karolinska Institutet, Stockholm, Sweden, **8** Department of Medical Oncology, Animus Kyanus Stavros General Clinic, Larissa, Greece
