# Supplementary material for: Effect of body tissue composition on the outcome of patients with metastatic non-small cell lung cancer treated with PD-1/PD-L1 inhibitors
Source: PLoS One. 2023 Feb 10;18(2):e0277708. doi: 10.1371/journal.pone.0277708 (PMC9916610; doi:10.1371/journal.pone.0277708)
Supplement: S1 File — (DOCX) [file pone.0277708.s001.docx]

**Supplementary data**

**Figure S1**: Scatter-plots demonstrating the correlation between baseline BMI values and **A.** baseline VFI values, **B.** baseline SFI values **C.** baseline LSMI values **D.** baseline IMFI values

**A. B.**

**
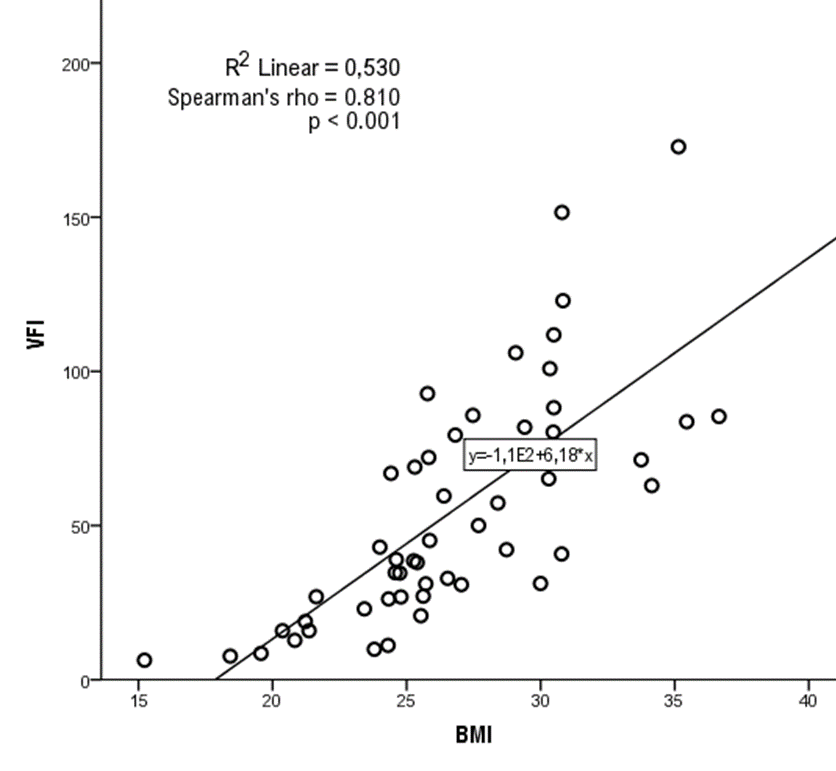

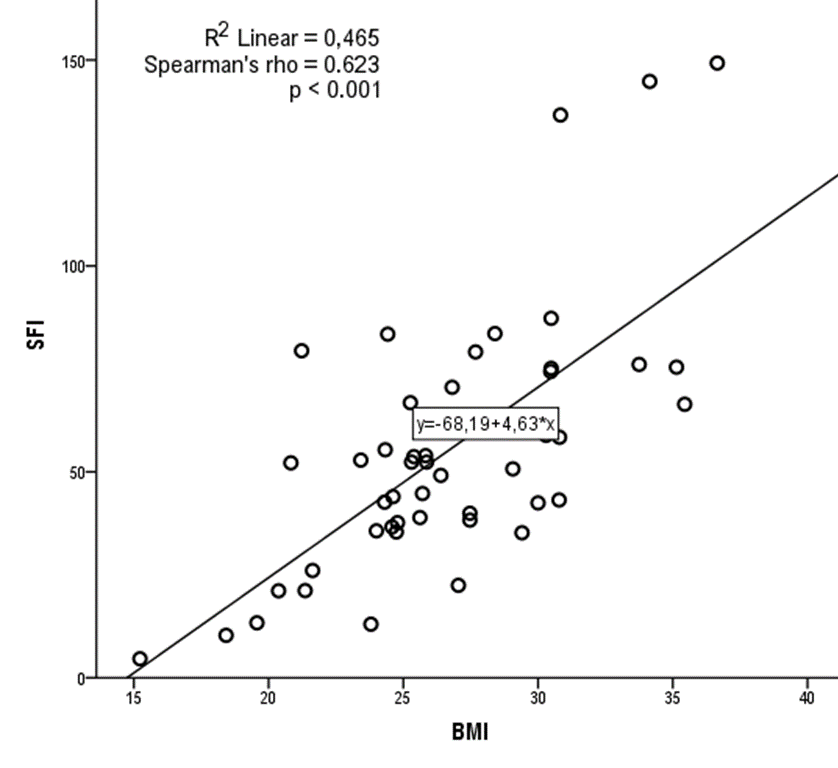
**

**C. D.**


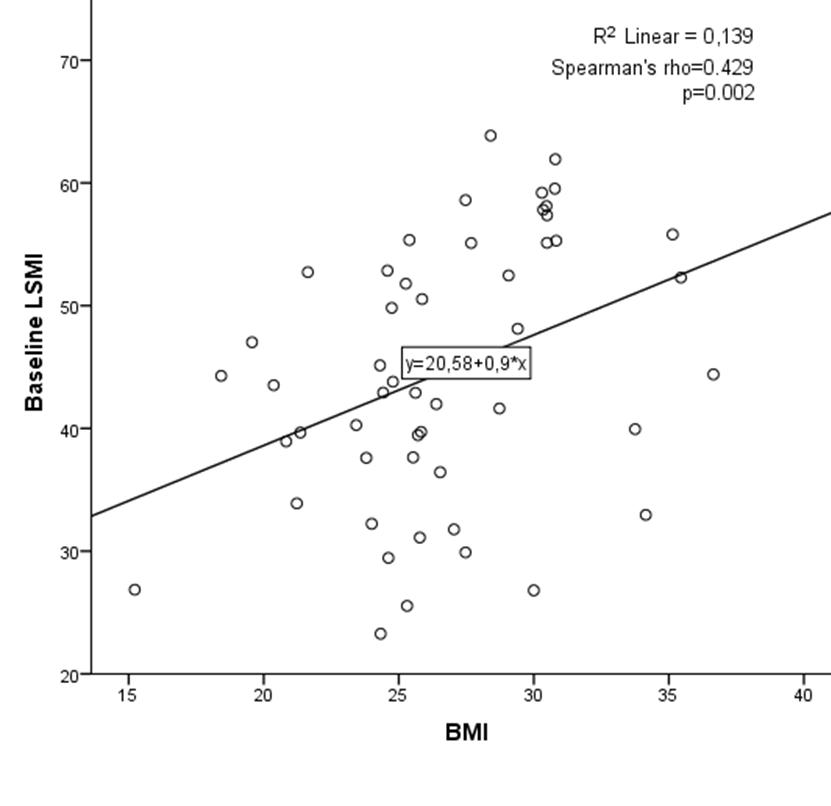

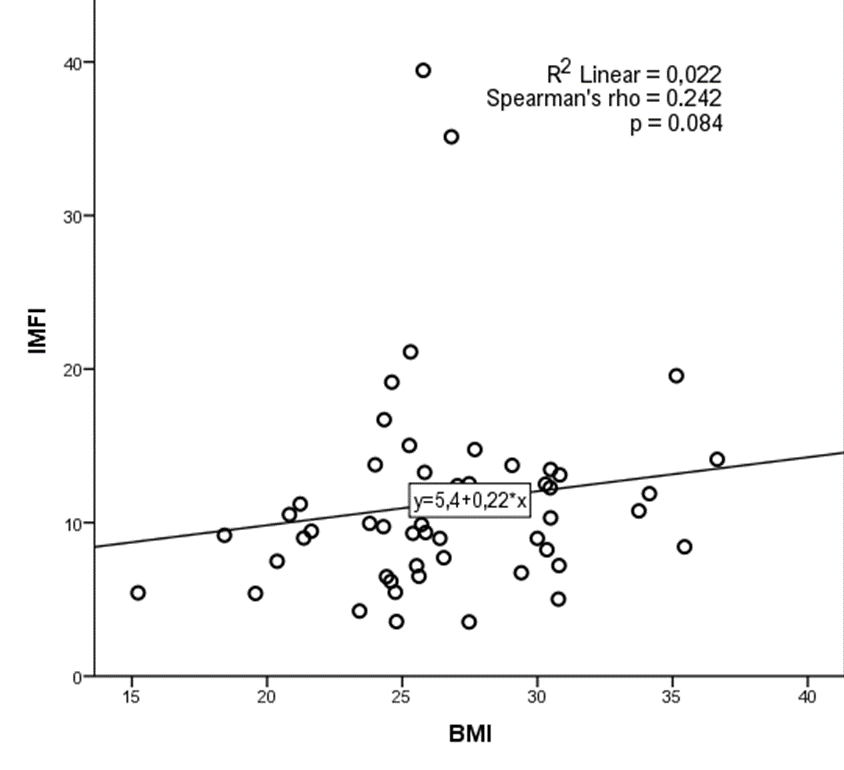


**Abbreviations:** BMI=Body mass index, LSMI: Lumbar skeletal muscle index (At the level of 3^rd^ lumbar vertebra), IMFI=Intramuscular Fat Index (At the level of 3^rd^ lumbar vertebra), VFI=Visceral Fat Index (At the level of 3^rd^ lumbar vertebra), SFI=Subcutaneous Fat Index (At the level of 3^rd^ lumbar vertebra)

**Figure S2:** Scatter-plots demonstrating the correlation between baseline LMSI values and **A.** baseline IMFI values, **B.** baseline VFI values **C.** baseline SFI values

1. **B.**

**
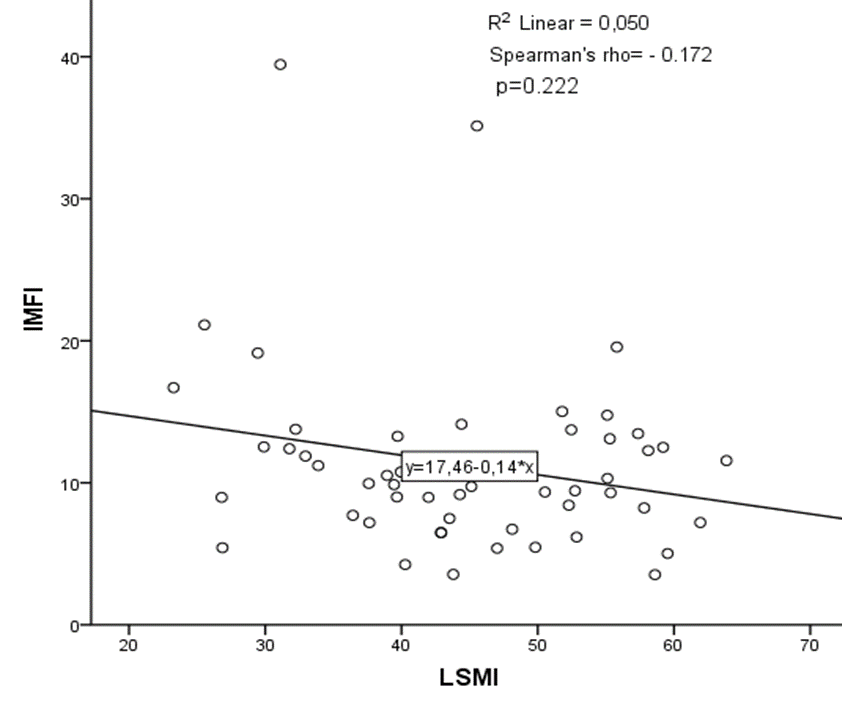

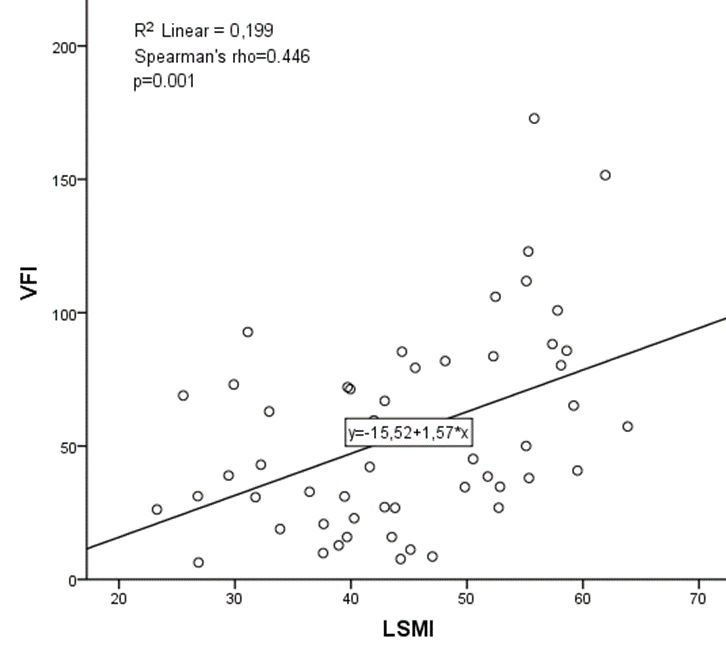
**

**C.**


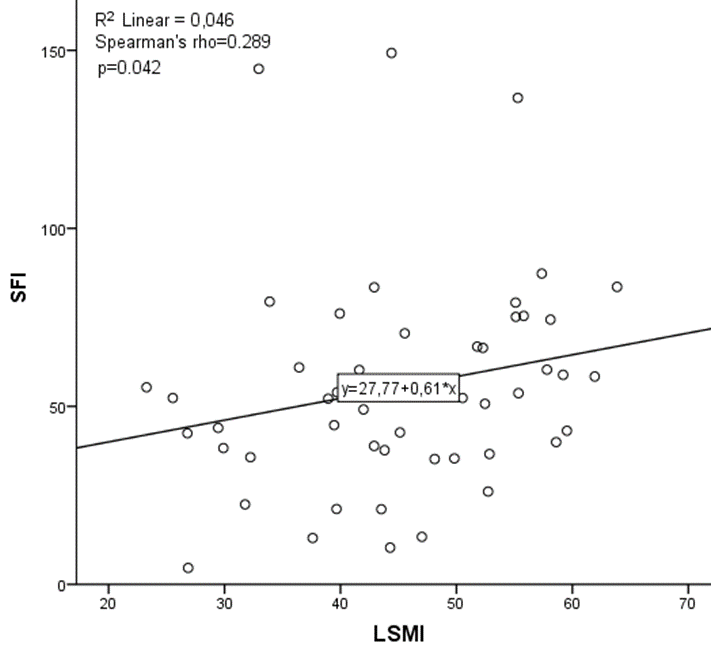


**Abbreviations:** LSMI: Lumbar skeletal muscle index (At the level of 3^rd^ lumbar vertebra), IMFI=Intramuscular Fat Index (At the level of 3^rd^ lumbar vertebra), VFI=Visceral Fat Index (At the level of 3^rd^ lumbar vertebra), SFI=Subcutaneous Fat Index (At the level of 3^rd^ lumbar vertebra)

**Figure S3:** Box-plots demonstrating the differential distributions (Mann Whitney U test) of **A.** Baseline* BMI values between responders and non-responders **B.** IMFI values between responders and non-responders **C.** VFI values between responders and non-responders **D.** LSMI values between responders and non-responders **E.** IMFI values in patients who achieved disease control (CR or PR or SD) versus those who experienced PD **F.** LSMI values of individuals who achieved disease control versus those who had disease progression.

1. **B. C.**

**
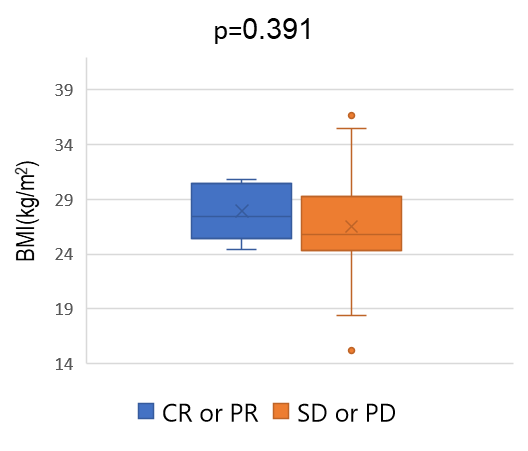

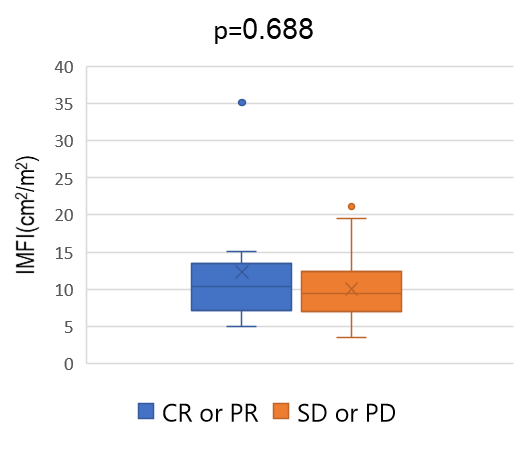

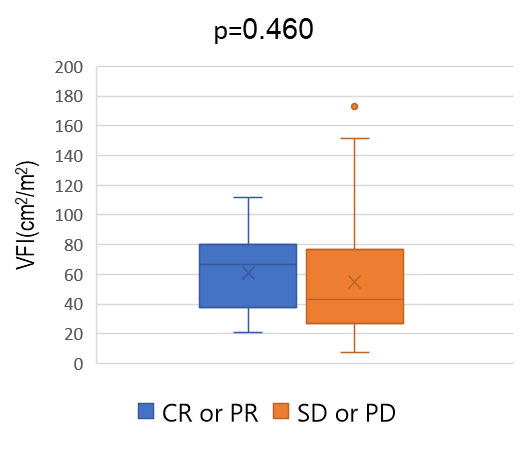
**

**D. E. F.**

**
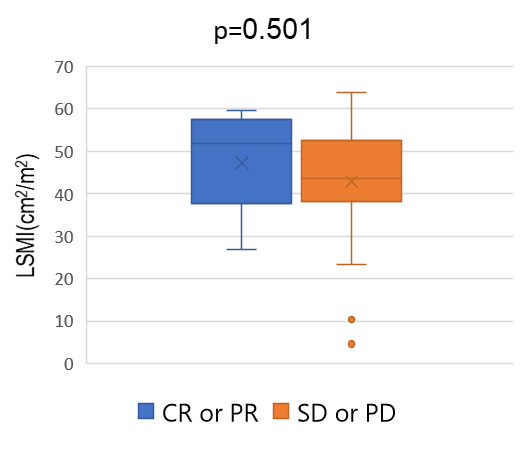

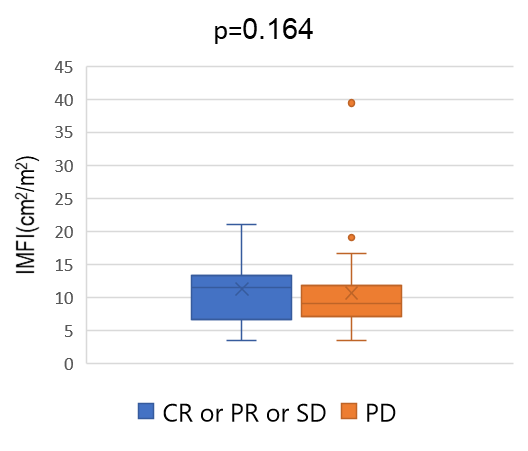
**
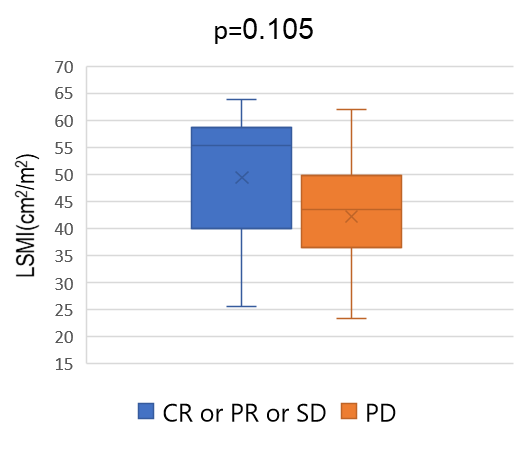


**Abbreviations:** BMI=Body mass index, LSMI: Lumbar skeletal muscle index (At the level of 3^rd^ lumbar vertebra), IMFI=Intramuscular Fat Index (At the level of 3^rd^ lumbar vertebra), VFI=Visceral Fat Index (At the level of 3^rd^ lumbar vertebra), SFI=Subcutaneous Fat Index (At the level of 3^rd^ lumbar vertebra), CR: Complete response, PR: Partial response, SD: Stable disease, PD: Progressive disease

*** Baseline:** At the beginning of immunotherapy

**Table S1:** Effect of the studied variables on objective response rate (ORR).

| **Variable** | **N=52** | **CR or PR** | **SD or PD** | ***P* value**  **(chi-square test, 95% CI)** |
| --- | --- | --- | --- | --- |
| **Age** |  | | | |
| < 70 years old | 31 | 7 | 25 | 0.918 |
| ≥ 70 years old | 21 | 5 | 16 |  |
| **Gender** |  | | | |
| Male | 43 | 10 | 33 | 0.947 |
| Female | 9 | 2 | 7 |  |
| **Performance status** |  | | | |
| 0-1 | 41 | 10 | 31 | 0.664 |
| 2 | 9 | 2 | 11 |  |
| **Histology** |  |  | | |
| Non-squamous | 30 | 7 | 23 | 0.959 |
| Squamous | 22 | 5 | 17 |  |
| **Brain metastases** |  | | | |
| Yes | 10 | 1 | 9 | 0.275 |
| No | 42 | 11 | 31 |  |
| **Liver metastases** |  | | | |
| Yes | 14 | 3 | 11 | 0.864 |
| No | 38 | 9 | 29 |  |
| **Bone metastases** |  | | | |
| Yes | 15 | 4 | 11 | 0.696 |
| No | 37 | 8 | 29 |  |
| **PD-L1 status** | **N=33** | | | |
| < 1 % | 10 | 2 | 8 | 0.708 |
| ≥ 1 % | 23 | 6 | 17 |  |
| **Baseline albumin levels** | **N=47** | | | |
| < 3.5 g/dl | 6 | 0 | 6 | 0.202 |
| ≥ 3.5 g/dl | 41 | 9 | 32 |  |
| **BMI** |  | | | |
| < 25 kg/m^2^ | 18 | 2 | 16 | 0.136 |
| ≥ 25 kg/m^2^ | 34 | 10 | 24 |  |
| **Baseline LSMI** |  | | | |
| < LNL | 36 | 6 | 30 | 0.100 |
| ≥ LNL | 16 | 6 | 10 |  |
| **Baseline IMFI** |  | | | |
| Low | 26 | 5 | 21 | 0.510 |
| High | 26 | 7 | 19 |  |
| **Baseline VFI** |  | | | |
| Low | 26 | 6 | 20 | 1.000 |
| High | 26 | 6 | 20 |  |
| **Baseline SFI** | **N=50** | | | |
| Low | 25 | 3 | 22 | 0.088 |
| High | 25 | 8 | 17 |  |

**Abbreviations:** BMI=Body mass index; SD=Standard deviation; LSMI=Lumbar skeletal muscle index (At the level of 3^rd^ lumbar vertebra), LNL: Lower normal limit, 55 cm^2^/m^2^ for males and 39 cm^2^/m^2^ for females; IMFI=Intramuscular Fat Index (At the level of 3^rd^ lumbar vertebra); VFI=Visceral Fat Index (At the level of 3^rd^ lumbar vertebra); SFI=Subcutaneous Fat Index (At the level of 3^rd^ lumbar vertebra)

**Table S2:** Log-rank test on the effect of the studied variables on PFS and OS.

|  | **Median PFS**  **(Months)** | **p value**  **(log-rank test)** | **Median OS**  **(Months)** | **p value**  **(log-rank test)** |
| --- | --- | --- | --- | --- |
| All patients (n=52) |  |  |  |  |
| **Age** |  |  |  |  |
| < 70 years old | 4.00 | 0.754 | 9.43 | 0.512 |
| ≥ 70 years old | 7.33 |  | 13.37 |  |
| **Gender** |  |  |  |  |
| Male | 4.80 | 0.104 | 11.23 | 0.370 |
| Female | 1.53 |  | 6.77 |  |
| **Performance Status** |  |  |  |  |
| 0-1 | 4.80 | 0.360 | 12.70 | 0.140 |
| 2 | 3.50 |  | 5.27 |  |
| **Histology** |  |  |  |  |
| Squamous | 5.77 | 0.222 | 10.80 | 0.812 |
| Non-squamous | 2.57 |  | 9.90 |  |
| **BMI** |  |  |  |  |
| < 25 kg/m^2^ | 1.77 | 0.196 | 3.77 | 0.175 |
| 25 kg/m^2^ ≤ BMI < 30 kg/m^2^ | 6.30 |  | 10.30 |  |
| BMI ≥ 30 kg/m^2^ | 7.33 |  | 14.03 |  |
| **Line of treatment of ICI administration** |  |  |  |  |
| 1^st^ line | 7.33 | 0.088 | Not reached | **0.005** |
| 2^nd^ or later lines | 4.67 |  | 9.43 |  |
| **Brain metastases** |  |  |  |  |
| Yes | 1.57 | **0.006** | 4.80 | 0.083 |
| No | 4.93 |  | 12.70 |  |
| **Bone metastases** |  |  |  |  |
| Yes | 4.70 | 0.983 | 10.80 | 0.638 |
| No | 4.67 |  | 10.33 |  |
| **Liver metastases** |  |  |  |  |
| Yes | 1.53 | 0.120 | 3.77 | 0.059 |
| No | 4.80 |  | 12.70 |  |
| **Baseline albumin levels** |  |  |  |  |
| < 3.5 g/dl | 1.70 | **0.011** | 1.70 | **0.001** |
| ≥ 3.5 g/dl | 4.80 |  | 11.23 |  |
| **PD-L1 levels** |  |  |  |  |
| < 1% | 2.57 | 0.786 | 5.27 | 0.290 |
| ≥ 1% | 4.67 |  | 11.23 |  |
| **Baseline LSMI** |  |  |  |  |
| < LNL | 3.00 | **0.040** | 6.37 | **0.009** |
| ≥ LNL | 7.33 |  | Not reached |  |
| **Baseline IMFI** |  |  |  |  |
| Low | 3.03 | 0.647 | 10.80 | 0.229 |
| High | 4.80 |  | 12.70 |  |
| **Baseline VFI** |  |  |  |  |
| Low | 3.03 | 0.975 | 6.37 | 0.231 |
| High | 4.93 |  | 11.23 |  |
| **Baseline SFI** |  |  |  |  |
| Low | 2.97 | 0.135 | 5.43 | **0.020** |
| High | 5.77 |  | 14.03 |  |

**Abbreviations:** BMI=Body mass index, ICI=Immune checkpoint inhibitor, PD-L1=Programmed death ligand-1, LSMI=Lumbar skeletal muscle index (cm2/m2) (At the level of 3^rd^ lumbar vertebra), LNL=Lower normal limits, 55 cm^2^/m^2^ for males and 39 cm^2^/m^2^ for females, IMFI=Intramuscular fat index (cm^2^/m^2^) (At the level of 3rd lumbar vertebra), Low: Below gender specific median value, VFI=Visceral fat index (cm^2^/m^2^) (At the level of 3rd lumbar vertebra), Low: Below gender specific median value, SFI=Subcutaneous fat index (cm^2^/m^2^) (At the level of 3rd lumbar vertebra), Low: Below gender specific median value

**Figure S4:** Log-rank test demonstrating the different survival outcomes of **A.** The patients in our cohort according to the combination of their baseline*^1^ SFI and LSMI values **B.** Between patients with high^*2^ SFI and LMSI ≥ LNL^*3^ and patients with high SFI and LMSI < LNL.

**A.**


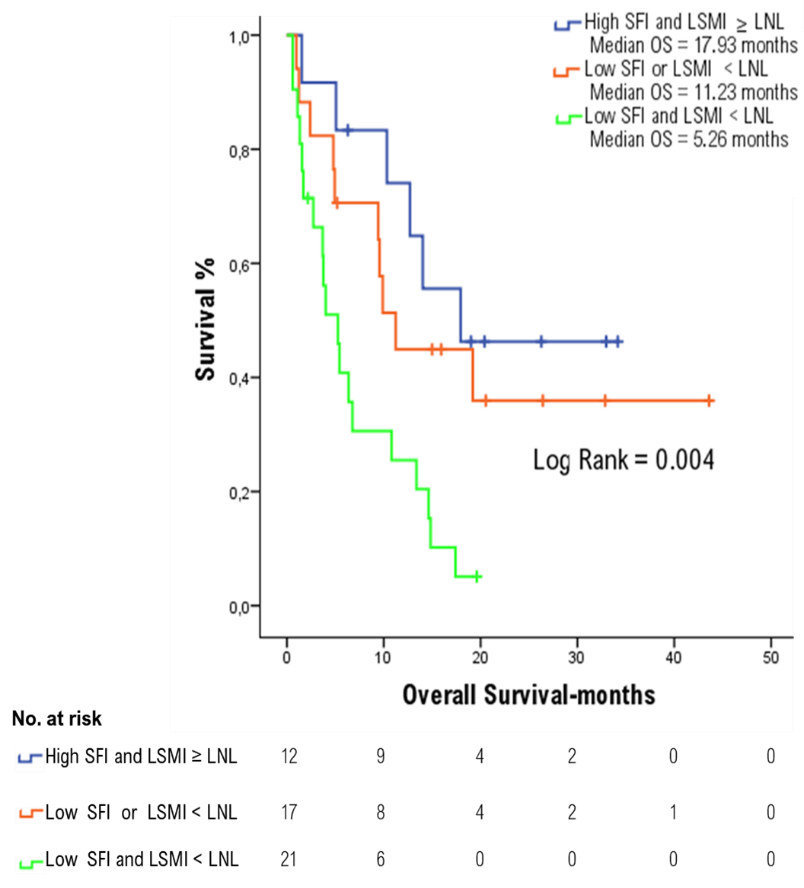


**B.**


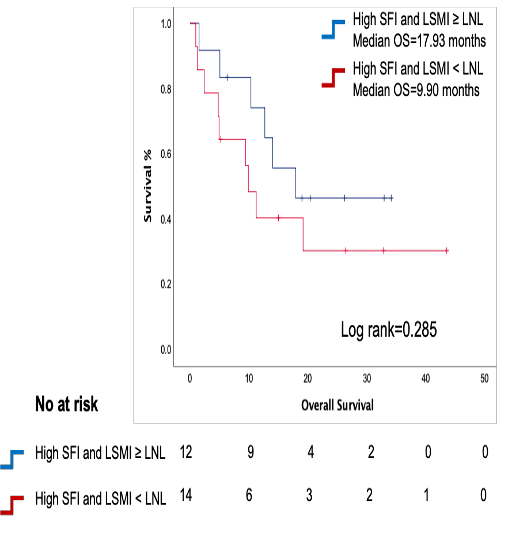


**Abbreviations:** LSMI=Lumbar skeletal muscle index (cm2/m2) (At the level of 3^rd^ lumbar vertebra), SFI=Subcutaneous fat index (cm^2^/m^2^) (At the level of 3rd lumbar vertebra), OS=Overall survival

*^1^ **Baseline:** At the beginning of immunotherapy

*^2^ **Low:** Below gender specific median value; High: Above gender specific median value

*^3^ **LNL:** Lower normal limit, 55 cm^2^/m^2^ for males and 39 cm^2^/m^2^ for females

**Figure S5:** Scatter-plot demonstrating the overall survival values of the patients according to their baseline SFI. Univariate Cox Regression analysis for OS for SFI as continuous variable HR=**0.983** (0.970-0.997), p=**0.014**. Gender was used as a stratification factor.


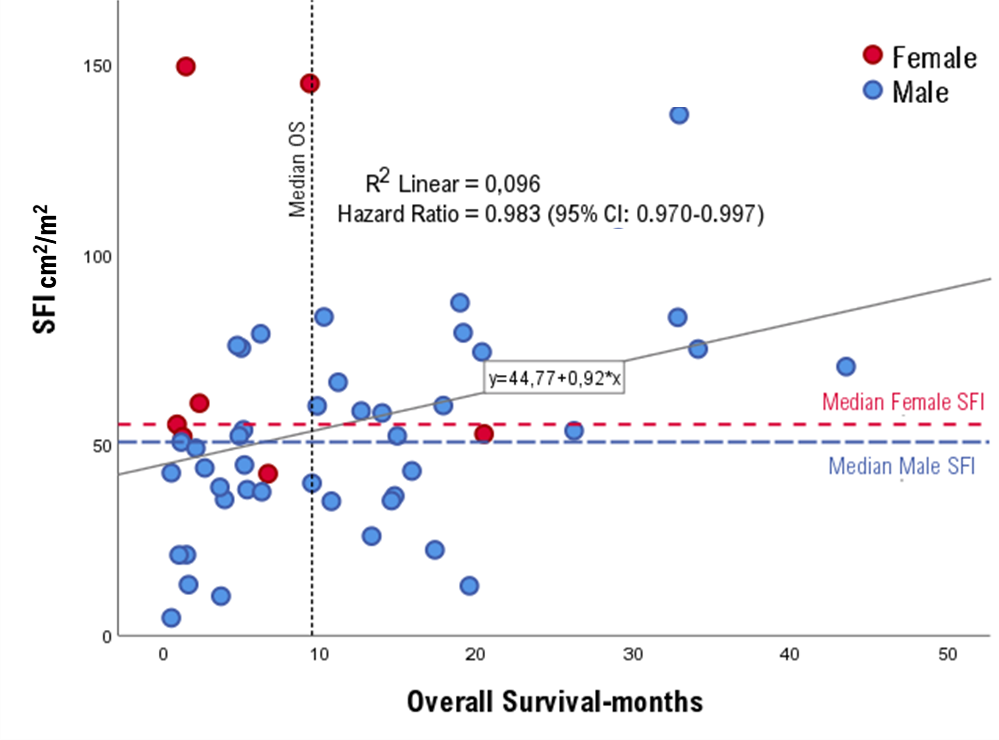


**Abbreviations:** SFI=Subcutaneous fat index (cm^2^/m^2^) (At the level of 3rd lumbar vertebra), OS=Overall survival

**Table S3:** Univariate analysis using Cox regression method investigating the hazard ratios of the analyzed categorical covariates on PFS and OS.

| ***COX REGRESSION*** | **PFS** | | **OS** | |
| --- | --- | --- | --- | --- |
| *UNIVARIATE ANALYSIS* | HR  (95% Confidence Intervals) | p value | HR  (95% Confidence Intervals) | p value |
| Age ≥ 70 years old | 0.91 (0.496-1.663) | 0.755 | 0.80 (0.407-1.568) | 0.514 |
| Performance status 2 | 1.39 (0.684-2.823) | 0.363 | 1.76 (0.821-3.773) | 0.146 |
| Female gender | 1.89 (0.864-4.141) | 0.111 | 1.50 (0.616-3.636) | 0.373 |
| Squamous histology | 0.69 (0.374-1.262) | 0.226 | 0.92 (0.473-1.798) | 0.812 |
| BMI < 25 kg/m^2^ | 1.76 (0.942-3.282) | 0.076 | 1.85 (0.942-3.618) | 0.074 |
| Brain metastases | **2.71** (1.299-5.667) | **0.008** | 2.02 (0.898-4.529) | 0.089 |
| Liver metastases | 1.67 (0.868-3.213) | 0.125 | 1.94 (0.962-3.905) | 0.064 |
| Bone metastases | 0.99 (0.517-1.905) | 0.983 | 1.18 (0.588-2.378) | 0.639 |
| PD-L1 < 1% | 1.73 (0.474-2.677) | 0.787 | 1.64 (0.652-4.081) | 0.295 |
| Baseline LSMI < LLN | **2.03** (1.018-4.032) | **0.044** | **2.90** (1.261-6.667) | **0.012** |
| Low baseline IMFI | 1.15 (0.631-2.096) | 0.648 | 1.50 (0.770-2.931) | 0.232 |
| Low baseline VFI | 1.03 (0.551-1.848) | 0.975 | 1.50 (0.769-2.919) | 0.235 |
| Low baseline SFI | 1.59 (0.860-2.925) | 0.140 | **2.20** (1.114-4.333) | **0.023** |

**Abbreviations:** BMI=Body mass index, PD-L1=Programmed death ligand-1, LSMI=Lumbar skeletal muscle index (cm^2^/m^2^), LNL: 55 cm^2^/m^2^ for males and 39 cm^2^/m^2^ for females, IMFI=Intramuscular fat index (cm^2^/m^2^). Low: Below gender specific median value, VFI=Visceral fat index (cm^2^/m^2^). Low: Below gender specific median value, SFI=Subcutaneous fat index (cm^2^/m^2^). Low: Below gender specific median value
